# Perioperative immune trajectories in severe scoliosis surgery: a retrospective study of 2,736 patients undergoing corrective surgery for severe scoliosis

**DOI:** 10.3389/fimmu.2026.1835306

**Published:** 2026-07-06

**Authors:** Tao Chen, Yong Chen, Qiang Li, Wenjing Zhang, Jindong Yang, Chun Pu, Yinxiao Peng, Qin Fei, Qiang Fu

**Affiliations:** 1 Department of Anesthesiology, The Third People’s Hospital of Chengdu, Chengdu, Sichuan, China; 2 Health Management Center, The Third People’s Hospital of Chengdu, Chengdu, Sichuan, China; 3 Department of Orthopedics, The Third People’s Hospital of Chengdu, Chengdu, Sichuan, China

**Keywords:** IL-6, immune trajectory, latent class mixed modeling, lymphopenia, monocyte HLA-DR, neutrophil-to-lymphocyte ratio, perioperative immunity, postoperative infection

## Abstract

**Background:**

Severe scoliosis surgery is accompanied by substantial perioperative immune perturbation, yet the heterogeneity of postoperative immune recovery and its relationship with adverse clinical outcomes remain insufficiently defined. Methods: We performed a single-center retrospective longitudinal cohort study in one of China’s largest care centers for severe scoliosis, including 2,736 patients who underwent corrective surgery for severe scoliosis between January 1, 2018, and December2024, 2024 and had serial immune measurements at preoperative baseline, 6 h, postoperative day (POD) 1, POD3, POD5, and discharge. Latent class mixed modeling based on absolute lymphocyte count, neutrophil-to-lymphocyte ratio, IL-6, and monocyte HLA-DR was used to identify perioperative immune trajectory phenotypes. The primary endpoint was major postoperative infection within 30 days. Models were adjusted for demographic, deformity-related, operative, and perioperative-management covariates, including surgical approach, steroid exposure, and opioid requirement.

**Results:**

Three distinct immune trajectory phenotypes were identified: adaptive recovery (n = 1,290, 47.1%), intermediate recovery (n = 903, 33.0%), and persistent dysregulated immunity (n = 543, 19.8%). Compared with the other two classes, the persistent dysregulated class was characterized by lower perioperative lymphocyte counts, higher neutrophil-to-lymphocyte ratio and IL-6 levels, more sustained monocyte HLA-DR suppression, higher C-reactive protein levels, lower CD4/CD8 ratio, and delayed albumin recovery. Major postoperative infection occurred in 8.3%, 16.1%, and 24.5% of the three classes, respectively (P <0.001), while prolonged postoperative length of stay occurred in 7.1%, 25.5%, and 66.9%, respectively (P <0.001). After multivariable adjustment, intermediate recovery and persistent dysregulated immunity remained associated with major infection (adjusted odds ratio [aOR] 2.06, 95% CI 1.54-2.76, and 3.31, 95% CI 2.22-4.94, respectively; both P <0.001). Corresponding associations with prolonged length of stay were 3.71 (95% CI 2.80-4.91) and 17.84 (95% CI 12.34-25.79), respectively (both P <0.001). The infection model showed moderate discrimination (area under the receiver operating characteristic curve, 0.668).

**Conclusion:**

Severe scoliosis surgery is followed by clinically meaningful perioperative immune recovery phenotypes. These trajectories are associated with infection and prolonged hospitalization but should be interpreted as dynamic markers reflecting both host response and perioperative stress rather than as purely causal immune endotypes.

## 1 Introduction

Severe spinal deformities, particularly severe scoliosis, remain a major challenge in pediatric and adult spinal surgery. Early-onset scoliosis can progress rapidly and is associated with thoracic insufficiency and cardiorespiratory morbidity (1). The epidemiology and clinical burden vary by etiology: adolescent idiopathic scoliosis is relatively common, congenital scoliosis is less frequent, and neuromuscular scoliosis is often progressive and associated with impaired sitting balance and reduced vital capacity (2–4). In cerebral palsy, severe curves occur mainly in non-ambulatory patients and can substantially affect pulmonary function, seating tolerance, and quality of life (5–7). These features make severe scoliosis a clinically vulnerable population in which postoperative risk is shaped by both deformity severity and host physiological reserve.

Corrective spinal surgery for severe deformity, while often necessary to halt progression, improve posture, and potentially enhance respiratory mechanics, is among the most complex and invasive procedures in orthopedics (8, 9). The surgical intervention itself imposes a massive physiological insult. Procedures for severe rigid curves, often requiring advanced techniques like vertebral column resection or staged approaches with preoperative halo-pelvic traction, are characterized by prolonged operative times, significant blood loss, and substantial tissue trauma (9, 10). This complexity translates directly into a high rate of perioperative complications. Data from the Scoliosis Research Society Morbidity and Mortality database consistently show that spinal deformity surgery carries serious risks, with neuromuscular scoliosis (NMS) exhibiting the highest infection rates among all etiologies, ranging from 3.24% to 3.94% (11). Postoperative complications are diverse, encompassing respiratory failure, surgical site infections (SSI), neurological deficits, and gastrointestinal issues like postoperative ileus (12–14). The burden is particularly pronounced in patients with underlying conditions such as cerebral palsy, who incur significantly higher costs and longer hospital stays compared to those with idiopathic scoliosis, and face elevated risks of mortality, pneumonia, and urinary tract infections (15). This elevated risk profile underscores the critical need to understand the host-specific physiological responses that underpin the complications.

Major surgery triggers a highly coordinated yet potentially maladaptive physiological cascade known as the surgical stress response, which integrates neuroendocrine, immune, and metabolic pathways (16). This response is initiated by afferent neural signals and hormonal mediators, leading to the release of cortisol and catecholamines. A central component of this response is perioperative immune dysregulation, a biological framework characterized by a biphasic reaction: an initial systemic inflammatory activation followed frequently by a state of compensatory immunosuppression (16). The inflammatory phase is marked by a surge of pro-inflammatory cytokines, such as interleukin-6 (IL-6) and tumor necrosis factor-alpha (TNF-α), and the mobilization of innate immune cells like neutrophils (17, 18). Concurrently, the lymphocytic arm of the adaptive immune system often becomes suppressed, a phenomenon reflected in postoperative lymphopenia. Monocytes, key orchestrators of the immune response, can exhibit dysfunction, notably downregulation of Human Leukocyte Antigen DR (HLA-DR), impairing antigen presentation. This dysregulated state, while aimed at promoting healing, can paradoxically increase susceptibility to infections, impede wound healing, and contribute to organ dysfunction. In patients already burdened by conditions like neuromuscular disease, the attenuated protective physiological responses are further compromised, making perioperative optimization through a multidisciplinary approach essential yet exceptionally challenging (19, 20).

The clinical translation of this biological framework has led to intensive investigation of specific immune and inflammatory biomarkers as tools for risk stratification and early diagnosis of postoperative complications. The neutrophil-to-lymphocyte ratio (NLR), an easily calculable index reflecting the balance between innate inflammatory response and adaptive immune capacity, has emerged as a valid biomarker of systemic inflammation and stress (21). Elevated NLR values have demonstrated utility in predicting the development of sepsis after liver transplantation and are associated with surgical complications, including surgical site infection, in various procedural contexts (22, 23). Other pivotal markers include C-reactive protein (CRP), a classic acute-phase reactant whose dynamic postoperative rise is widely monitored, and interleukin-6 (IL-6), a key pro-inflammatory cytokine with diagnostic value in periprosthetic joint infections (24, 25). Procalcitonin (PCT) is also employed, though its interpretation can be confounded by factors such as certain antibiotic therapies (26). The predictive utility of the markers extends beyond infection; for example, postoperative increases in NLR and CRP have been independently linked to higher-grade complications in oncologic surgery (23). Systematic reviews affirm that CRP measured between postoperative days three and seven holds moderate to high predictive value for infection across surgical specialties (25). Still, the prevailing paradigm relies heavily on isolated measurements or limited serial snapshots, assessing biomarker levels at single predefined time points, which may not capture the dynamic and heterogeneous nature of individual immune recovery.

Within the specific context of scoliosis surgery, the application of this biomarker knowledge remains relatively nascent and fragmented. Existing research has effectively delineated the high incidence of complications, particularly in neuromuscular cohorts, and identified static risk factors such as etiology, non-ambulatory status, and preoperative pulmonary function (13, 27). Studies have reported on the effectiveness of local antibiotic strategies to reduce SSI and described complication profiles from large registries (28, 29). The profound impact of surgery on mortality, notably the reduction in respiratory-related deaths in CP patients with scoliosis who undergo surgical correction, highlights the critical intersection of surgical intervention and systemic physiology (30). Despite the advances, a significant knowledge gap persists. The current literature largely provides a cross-sectional view, reporting complication rates and analyzing static preoperative variables or single postoperative biomarker values. A detailed, longitudinal profiling of the immune trajectory throughout the entire perioperative journey in this high-stakes surgical population is conspicuously lacking. The immune response to the immense trauma of severe spinal reconstruction is likely not uniform but may consist of distinct phenotypic patterns of recovery or dysregulation that are poorly characterized by isolated biomarker assessments.

This gap supports a longitudinal approach to perioperative immune phenotyping. Patients undergoing major spinal reconstruction may not follow a uniform biomarker course; instead, they may cluster into reproducible patterns of inflammatory activation, lymphocyte depletion and recovery, and monocyte antigen-presentation capacity. These patterns may improve risk stratification beyond isolated biomarker measurements, although they should be interpreted cautiously because postoperative trajectories can reflect both biological host response and the magnitude of surgical stress (31, 32).

Therefore, to address the important unmet need for a dynamic, systems-level understanding of the host response in this vulnerable surgical population, we conducted a comprehensive longitudinal cohort study. The primary objective was to delineate distinct perioperative immune trajectory phenotypes using serial, multi-parametric immune measurements in patients undergoing corrective surgery for severe scoliosis. We further aimed to rigorously evaluate the association of the identified immune phenotypes with the risk of major postoperative infection within 30 days. Secondarily, we sought to examine the links between immune phenotypes and other clinically meaningful outcomes, including prolonged postoperative length of stay, 30-day hospital readmission, and 90-day reoperation. We hypothesized that patients would cluster into discrete immune recovery phenotypes based on their longitudinal profiles and that the phenotypes would be independently associated with significant differences in the risk of adverse postoperative outcomes, thereby providing a novel immunological framework for risk stratification and future interventional studies in complex spinal deformity surgery.

## 2 Methods

### 2.1 Study design and setting

We conducted a single-center retrospective longitudinal cohort study in the Department of Anesthesiology, the Third People’s Hospital of Chengdu. Historical perioperative data were retrieved from the electronic medical record, laboratory information system, anesthetic charting system, and postoperative follow-up registry for consecutive patients who underwent surgery for severe scoliosis between January 1, 2018, and December 31, 2024. The study was approved by the Ethics Committee of the Third People’s Hospital of Chengdu (approval number 2024-T-119), which waived the requirement for individual informed consent because the study used de-identified historical data and involved no direct patient contact.

### 2.2 Participants

Eligible patients were required to have severe scoliosis, defined *a priori* as a major Cobb angle of at least 70°, and to have undergone definitive corrective surgery with posterior-only or combined anterior-posterior reconstruction under general anesthesia. Surgical approach was abstracted from operative records and categorized as posterior-only single-stage correction or combined/staged reconstruction; three-column osteotomy, posterior column osteotomy, and halo-assisted staged correction were recorded when documented. Patients were included if serial perioperative immune measurements were available at all predefined time points: preoperative baseline, 6 hours after surgery, postoperative day 1, postoperative day 3, postoperative day 5, and discharge. Patients were excluded if they had non-severe scoliosis, reoperation within 24 hours of index surgery, documented active infection before surgery, age younger than 10 years, or an incomplete serial immune dataset after screening.

### 2.3 Data collection and variable definitions

Data were collected by structured historical retrieval using a prespecified data dictionary. Baseline variables included age, sex, body mass index, scoliosis etiology, major Cobb angle, baseline nutritional status, preoperative hypoalbuminemia, American Society of Anesthesiologists physical status, and revision status. Operative variables included surgical approach, fusion length, osteotomy, operative time, estimated blood loss, red blood cell transfusion requirement, perioperative steroid exposure, opioid requirement during the first 48 postoperative hours, and intensive care unit admission. Immune and inflammatory variables included absolute lymphocyte count, neutrophil-to-lymphocyte ratio, serum IL-6, monocyte HLA-DR expression, C-reactive protein, albumin, and CD4/CD8 ratio at each prespecified perioperative time point. Absolute lymphocyte count, neutrophil-to-lymphocyte ratio, IL-6, and monocyte HLA-DR were selected as the trajectory-defining variables because they jointly captured adaptive immune depletion, innate inflammatory activation, cytokine surge, and monocyte antigen-presentation impairment, while remaining implementable in routine retrospective clinical datasets. Procalcitonin was not used for trajectory derivation because it was not measured systematically at all six time points during the full study period; forcing its inclusion would have substantially reduced sample size and introduced indication bias related to suspected infection.

Severe scoliosis etiology was classified as idiopathic, congenital, neuromuscular, or syndromic/other. Preoperative hypoalbuminemia was defined as serum albumin <35 g/L before surgery. Revision surgery referred to any deformity correction performed after prior spinal instrumentation or prior deformity surgery. Red blood cell transfusion exposure was analyzed as total intraoperative and immediate postoperative units. Perioperative steroid exposure was defined as any intravenous dexamethasone, methylprednisolone, or hydrocortisone administered from anesthetic induction through POD1; cumulative dose was converted to dexamethasone equivalents when available. Opioid exposure was converted to intravenous morphine milligram equivalents for the first 48 postoperative hours.

Perioperative management followed a standardized institutional spine-surgery pathway during the study period. General anesthesia was maintained with propofol-remifentanil total intravenous anesthesia or low-dose volatile anesthetic when neurophysiological monitoring permitted. Tranexamic acid was used routinely unless contraindicated, and antibiotic prophylaxis consisted of cefazolin or cefuroxime with vancomycin substitution for beta-lactam allergy or methicillin-resistant Staphylococcus aureus risk. Postoperative analgesia used patient-controlled intravenous sufentanil or morphine with acetaminophen and nonsteroidal anti-inflammatory drugs when not contraindicated; intrathecal morphine was used selectively in adolescent idiopathic cases (33, 34). Steroids were not administered as an immune-modifying intervention but were used for antiemetic prophylaxis, airway edema, neurologic concern, or adrenal replacement. These variables were abstracted from anesthesia records and included in adjusted or sensitivity analyses when sufficiently complete.

### 2.4 Outcomes

The primary endpoint was major postoperative infection within 30 days, defined as a composite of surgical site infection, pneumonia, urinary tract infection, bloodstream infection, or sepsis documented in the medical record and treated clinically as an infectious complication. Surgical site infection required surgeon-documented superficial or deep infection, wound drainage with antimicrobial treatment, positive culture, radiographic evidence, or operative debridement. Pneumonia required compatible symptoms or oxygen requirement plus radiographic infiltrate and antimicrobial treatment. Urinary tract infection and bloodstream infection required compatible clinical documentation with positive culture or targeted antimicrobial treatment. Secondary endpoints were prolonged postoperative length of stay, defined *a priori* as length of stay at or above the cohort 75th percentile, 30-day readmission, and 90-day reoperation. Individual infectious complications were also analyzed descriptively.

### 2.5 Immune trajectory modeling

Immune trajectory classes were derived from serial measurements of absolute lymphocyte count, neutrophil-to-lymphocyte ratio, IL-6, and monocyte HLA-DR across the six perioperative time points using latent class mixed modeling. Before model fitting, each trajectory-defining marker was standardized to a z score to place biomarkers measured on different scales onto a common metric. One-class through four-class models were prespecified and compared using the Bayesian information criterion, entropy, posterior class-membership probabilities, and minimum class size. The final model was selected on the basis of the best overall balance of statistical fit, class interpretability, and a minimum class proportion of 5%. Each patient was then assigned to the class with the highest posterior probability. For visualization only, a composite immune dysregulation score was calculated at each time point by averaging the standardized values of neutrophil-to-lymphocyte ratio, IL-6, inverse absolute lymphocyte count, and inverse monocyte HLA-DR, such that higher values indicated greater immune dysregulation. Because discharge occurred at variable postoperative days, an exploratory early-window model using only preoperative, 6 h, POD1, and POD3 measurements was also fitted to evaluate whether risk stratification was possible before discharge.

### 2.6 Sample size estimation

Because the study was retrospective, sample size was estimated to evaluate whether the available cohort would provide stable trajectory classification and adequately powered outcome modeling rather than to determine enrollment prospectively. We assumed a 30-day major infection rate of 10% to 15% in the overall cohort, three latent immune classes with the smallest class comprising at least 15% of patients, and a clinically important adjusted odds ratio of 1.8 for major infection in a higher-risk immune class versus the reference class. Under a two-sided alpha of 0.05, a cohort of approximately 3,200 patients would provide more than 90% power to detect that effect size in multivariable logistic regression while preserving at least 10 outcome events per parameter for the prespecified adjustment set. Because trajectory derivation also requires stable repeated-measures estimation, we further required at least 750 patients in the smallest class with complete biomarker profiles across all perioperative windows. The final sample exceeded that threshold comfortably, allowing robust trajectory derivation, clinically interpretable class sizes, and adequately powered adjusted analyses for both the primary endpoint and the main secondary endpoint.

### 2.7 Statistical analysis

Continuous variables were summarized as mean ± standard deviation or median with interquartile range according to distribution, and categorical variables were summarized as number and percentage. Between-class comparisons used one-way analysis of variance or the Kruskal-Wallis test for continuous variables and the chi-square test for categorical variables. The association between immune trajectory class and perioperative immune dysregulation over time was visualized using the composite immune dysregulation score and tested using a class-by-time interaction model. Multivariable logistic regression was used to evaluate the independent associations of immune trajectory class with major postoperative infection and prolonged length of stay after adjustment for age, sex, body mass index, scoliosis etiology, major Cobb angle, revision surgery, surgical approach, osteotomy, operative time, red blood cell transfusion, perioperative steroid exposure, 48-hour opioid requirement, and preoperative hypoalbuminemia. Sex was included *a priori*, and trajectory-by-sex interaction terms were tested for the primary endpoint. Additional sensitivity analyses repeated the primary model after excluding discharge biomarkers from trajectory derivation and after adding ICU admission as a marker of immediate postoperative physiological severity. Model discrimination was summarized using the area under the receiver operating characteristic curve. Adjusted odds ratios were reported with 95% confidence intervals. All statistical tests were two-sided, and P <0.05 was considered statistically significant. Analyses were performed in R version 4.3.2 with the packages lcmm version 2.1.0, mice version 3.16.0, tableone version 0.13.2, and pROC version 1.18.5.

## 3 Results

### 3.1 Cohort assembly and immune trajectory classification

**Figure 1:**
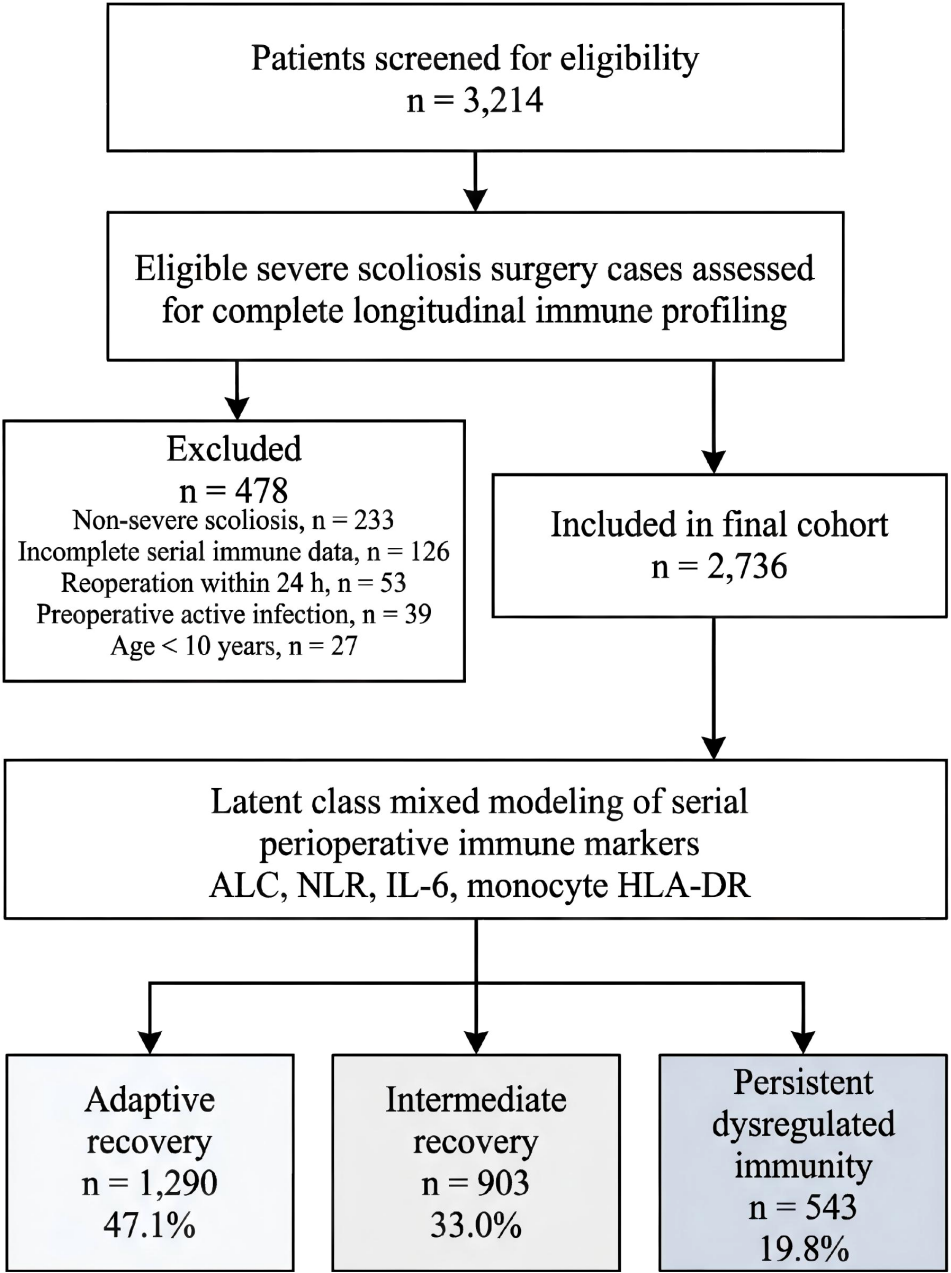
Study flowchart of cohort selection and immune trajectory classification. Flow diagram of patient screening, exclusions, final inclusion, and assignment to perioperative immune trajectory classes in a retrospective cohort of severe scoliosis surgery. Among 3,214 patients screened, 2,736 were included in the final analysis after exclusion of patients with non-severe scoliosis (*n =* 233), incomplete serial immune data after screening (*n =* 126), reoperation within 24 h (*n =* 53), preoperative active infection (*n =* 39), and age <10 years (*n =* 27). The final cohort was classified into three immune trajectory phenotypes: adaptive recovery (*n =* 1,290, 47.1%), intermediate recovery (*n =* 903, 33.0%), and persistent dysregulated immunity (*n =* 543, 19.8%).

A total of 3,214 patients were screened for eligibility during the study period. After exclusion of 478 patients, including 233 with non-severe scoliosis, 126 with incomplete serial immune measurements after screening, 53 who underwent reoperation within 24 hours, 39 with documented active infection before surgery, and 27 younger than 10 years, 2,736 patients were included in the final analysis. Using latent class mixed modeling of serial absolute lymphocyte count, neutrophil-to-lymphocyte ratio, IL-6, and monocyte HLA-DR across the six prespecified perioperative time points, a three-class solution was selected as the final trajectory model. In the model-selection process, the three-class model showed superior overall fit and clinical interpretability compared with the one- and two-class solutions, whereas the four-class model provided only marginal improvement in information criteria while generating an unstable small subgroup below the prespecified interpretability threshold. The final classes comprised 1,290 patients (47.1%) in the adaptive recovery class, 903 patients (33.0%) in the intermediate recovery class, and 543 patients (19.8%) in the persistent dysregulated class (**Figure 1**).

### 3.2 Baseline and operative characteristics

The mean age of the overall cohort was 18.3 years, and 1,826 patients (66.7%) were female. The adaptive recovery class was younger and had a higher proportion of idiopathic scoliosis, lower curve severity, shorter operative times, lower blood loss, fewer transfused red blood cell units, and less frequent ICU admission than the other two classes. By contrast, the persistent dysregulated class had the highest proportion of neuromuscular and syndromic etiologies, the greatest operative burden, the largest transfusion requirement, and the highest rate of postoperative ICU care. Posterior-only single-stage correction was the dominant surgical approach, but combined or staged reconstruction was more frequent in the persistent dysregulated class (8.1%, 10.7%, and 14.9% across adaptive, intermediate, and persistent classes, respectively; P <0.001). Perioperative steroid exposure occurred in 35.8% of the cohort and was similar across classes after accounting for surgical indication. Median 48-hour opioid exposure was 42, 48, and 56 morphine milligram equivalents across the three classes, respectively. Baseline and operative characteristics are summarized in **Table 1**.

**Table 1:** Baseline and operative characteristics by immune trajectory class (N = 2,736).

Characteristic	Adaptive recovery (*n =* 1,290)	Intermediate recovery (*n =* 903)	Persistent dysregulated (*n =* 543)	*P*
Age, y	17.0 ± 4.0	18.0 ± 4.7	21.3 ± 5.5	<0.001
Female, *n* (%)	888 (68.8)	578 (64.0)	325 (59.9)	0.001
BMI, kg/m²	19.9 ± 2.8	19.1 ± 2.8	18.4 ± 2.9	<0.001
Idiopathic etiology, *n* (%)	856 (66.4)	485 (53.7)	182 (33.5)	<0.001
Congenital etiology, *n* (%)	231 (17.9)	155 (17.2)	98 (18.0)	0.913
Neuromuscular etiology, *n* (%)	106 (8.2)	181 (20.0)	174 (32.0)	<0.001
Syndromic/other etiology, *n* (%)	97 (7.5)	82 (9.1)	89 (16.4)	<0.001
Cobb angle, °	83.8 ± 10.0	88.8 ± 11.9	94.0 ± 13.4	<0.001
Revision surgery, *n* (%)	106 (8.2)	113 (12.5)	96 (17.7)	<0.001
Osteotomy, *n* (%)	270 (20.9)	263 (29.1)	216 (39.8)	<0.001
Posterior-only single-stage correction, n (%)	1,186 (91.9)	806 (89.3)	462 (85.1)	<0.001
Combined or staged reconstruction, n (%)	104 (8.1)	97 (10.7)	81 (14.9)	<0.001
Fusion levels	11.6 ± 2.0	12.0 ± 2.0	12.9 ± 2.0	<0.001
Operative time, min	247.2 ± 50.7	271.6 ± 55.3	310.9 ± 57.0	<0.001
Blood loss, mL	821 (698-957)	1,056 (908-1,242)	1,429 (1,217-1,643)	<0.001
RBC units transfused	0.9 ± 0.8	1.3 ± 1.0	2.1 ± 1.3	<0.001
48-hour opioid exposure, morphine milligram equivalents	42 (31-55)	48 (36-63)	56 (42-71)	<0.001
ICU admission, *n* (%)	252 (19.5)	256 (28.4)	252 (46.4)	<0.001

### 3.3 Longitudinal immune and inflammatory profiles

**Figure 2:**
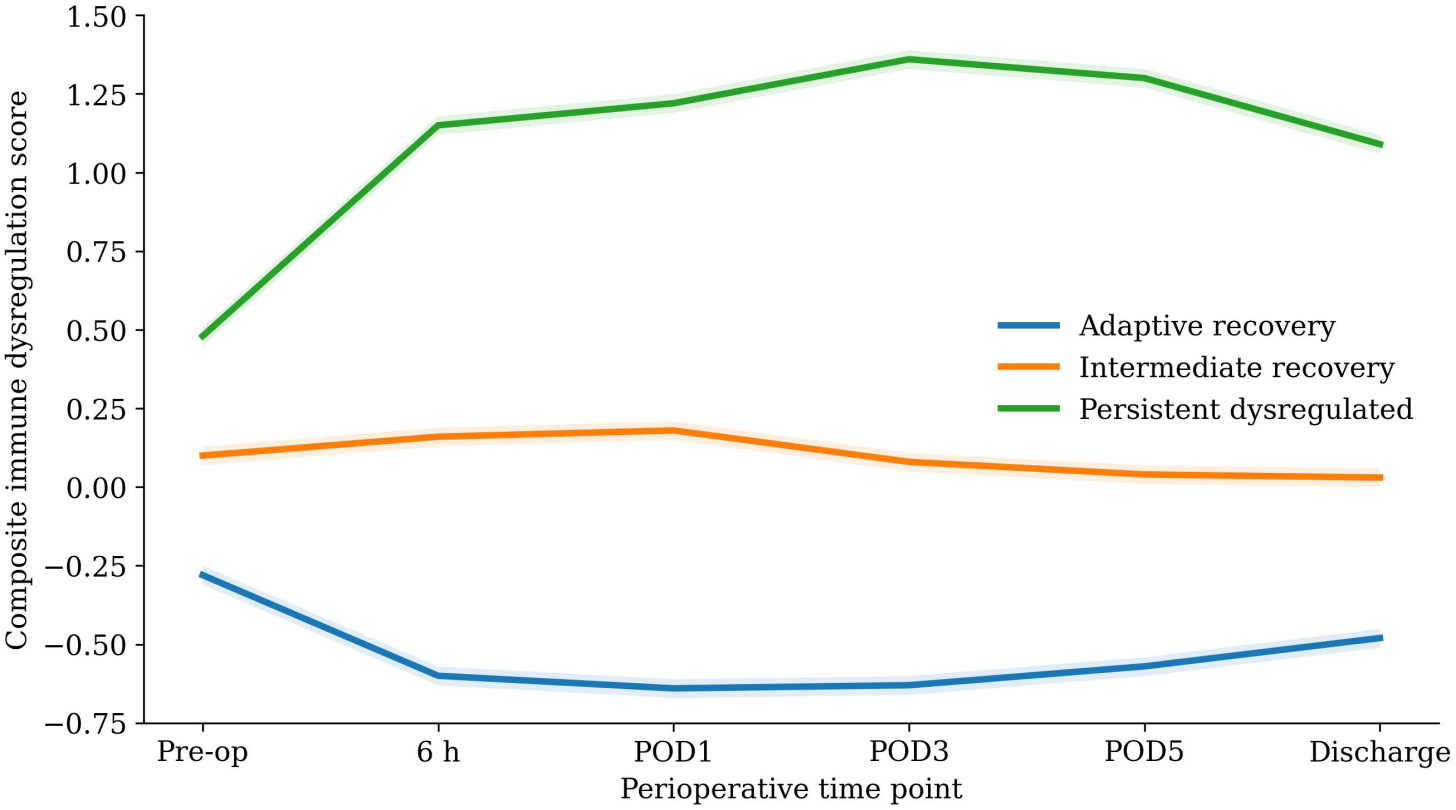
Composite perioperative immune dysregulation score according to immune trajectory class. Mean composite immune dysregulation score across six prespecified perioperative time points in patients classified into the adaptive recovery, intermediate recovery, and persistent dysregulated immune trajectory classes. The composite score was derived from standardized values of neutrophil-to-lymphocyte ratio, IL-6, inverse absolute lymphocyte count, and inverse monocyte HLA-DR, with higher values indicating greater immune dysregulation. Lines indicate class-specific means and shaded areas indicate 95% confidence intervals. The three classes showed sustained temporal separation, with the largest divergence from 6 h to POD5. The class-by-time interaction was significant (*P <* 0.001).

Longitudinal immune profiling demonstrated clear temporal separation among the three trajectory classes. The adaptive recovery class showed moderate postoperative inflammatory activation with preservation of immune recovery by discharge. The intermediate recovery class showed a higher cytokine and innate inflammatory response with delayed normalization. The persistent dysregulated class showed the deepest lymphopenic nadir, the highest NLR and IL-6 responses, persistently reduced monocyte HLA-DR, and incomplete recovery by discharge. Across all four selected perioperative checkpoints shown in **Table 2**, class differences were highly significant for absolute lymphocyte count, NLR, IL-6, monocyte HLA-DR, CRP, CD4/CD8 ratio, and albumin. When summarized using the composite immune dysregulation score, the three classes remained distinctly separated over time, with the strongest divergence from postoperative day 1 through postoperative day 5, as shown in **Figure 2**.

**Table 2:** Serial immune and inflammatory markers at selected perioperative time points.

Marker and time point	Adaptive recovery	Intermediate recovery	Persistent dysregulated	*P*
Absolute lymphocyte count, ×10^9^/L				
Preoperative	1.93 ± 0.29	1.73 ± 0.28	1.54 ± 0.27	<0.001
POD1	0.94 ± 0.29	0.75 ± 0.27	0.57 ± 0.28	<0.001
POD3	1.24 ± 0.28	0.96 ± 0.28	0.64 ± 0.27	<0.001
Discharge	1.79 ± 0.28	1.47 ± 0.29	1.04 ± 0.28	<0.001
Neutrophil-to-lymphocyte ratio				
Preoperative	2.50 ± 2.06	3.01 ± 2.23	3.73 ± 2.30	<0.001
POD1	11.16 ± 2.43	14.69 ± 2.38	19.75 ± 2.32	<0.001
POD3	6.77 ± 2.34	9.25 ± 2.40	15.08 ± 2.47	<0.001
Discharge	3.15 ± 2.21	4.40 ± 2.44	7.57 ± 2.31	<0.001
IL-6, pg/mL				
Preoperative	11.91 ± 14.58	13.50 ± 15.20	15.11 ± 16.61	0.001
POD1	80.95 ± 22.30	118.14 ± 22.26	166.63 ± 22.28	<0.001
POD3	38.84 ± 21.47	60.61 ± 22.99	108.59 ± 22.93	<0.001
Discharge	14.65 ± 16.02	18.12 ± 17.92	29.72 ± 20.70	<0.001
Monocyte HLA-DR, %				
Preoperative	81.91 ± 6.62	78.25 ± 6.52	74.70 ± 6.35	<0.001
POD1	63.34 ± 6.37	54.97 ± 6.66	45.09 ± 6.66	<0.001
POD3	70.04 ± 6.20	61.41 ± 6.49	46.65 ± 6.56	<0.001
Discharge	80.98 ± 6.73	74.21 ± 6.38	59.94 ± 6.39	<0.001
CRP, mg/L				
Preoperative	8.25 ± 10.56	9.01 ± 10.55	8.97 ± 11.03	0.229
POD1	68.15 ± 15.41	83.63 ± 14.74	102.10 ± 15.58	<0.001
POD3	91.43 ± 15.19	112.19 ± 15.00	137.63 ± 14.26	<0.001
Discharge	18.55 ± 13.15	29.07 ± 15.09	51.02 ± 14.61	<0.001
CD4/CD8 ratio				
Preoperative	1.73 ± 0.21	1.63 ± 0.22	1.57 ± 0.21	<0.001
POD1	1.37 ± 0.22	1.27 ± 0.22	1.19 ± 0.23	<0.001
POD3	1.47 ± 0.22	1.36 ± 0.22	1.22 ± 0.22	<0.001
Discharge	1.70 ± 0.23	1.57 ± 0.22	1.40 ± 0.22	<0.001
Albumin, g/L				
Preoperative	41.34 ± 2.39	40.10 ± 2.24	39.14 ± 2.27	<0.001
POD1	33.90 ± 2.46	32.58 ± 2.42	31.48 ± 2.39	<0.001
POD3	34.60 ± 2.41	32.85 ± 2.30	31.17 ± 2.30	<0.001
Discharge	39.03 ± 2.41	37.23 ± 2.45	34.85 ± 2.37	<0.001

As illustrated in **Figure 2**, the adaptive recovery class returned toward the lowest dysregulation burden by discharge, whereas the persistent dysregulated class remained clearly elevated across all postoperative measurements.

### 3.4 Postoperative outcomes according to immune trajectory class

**Figure 3:**
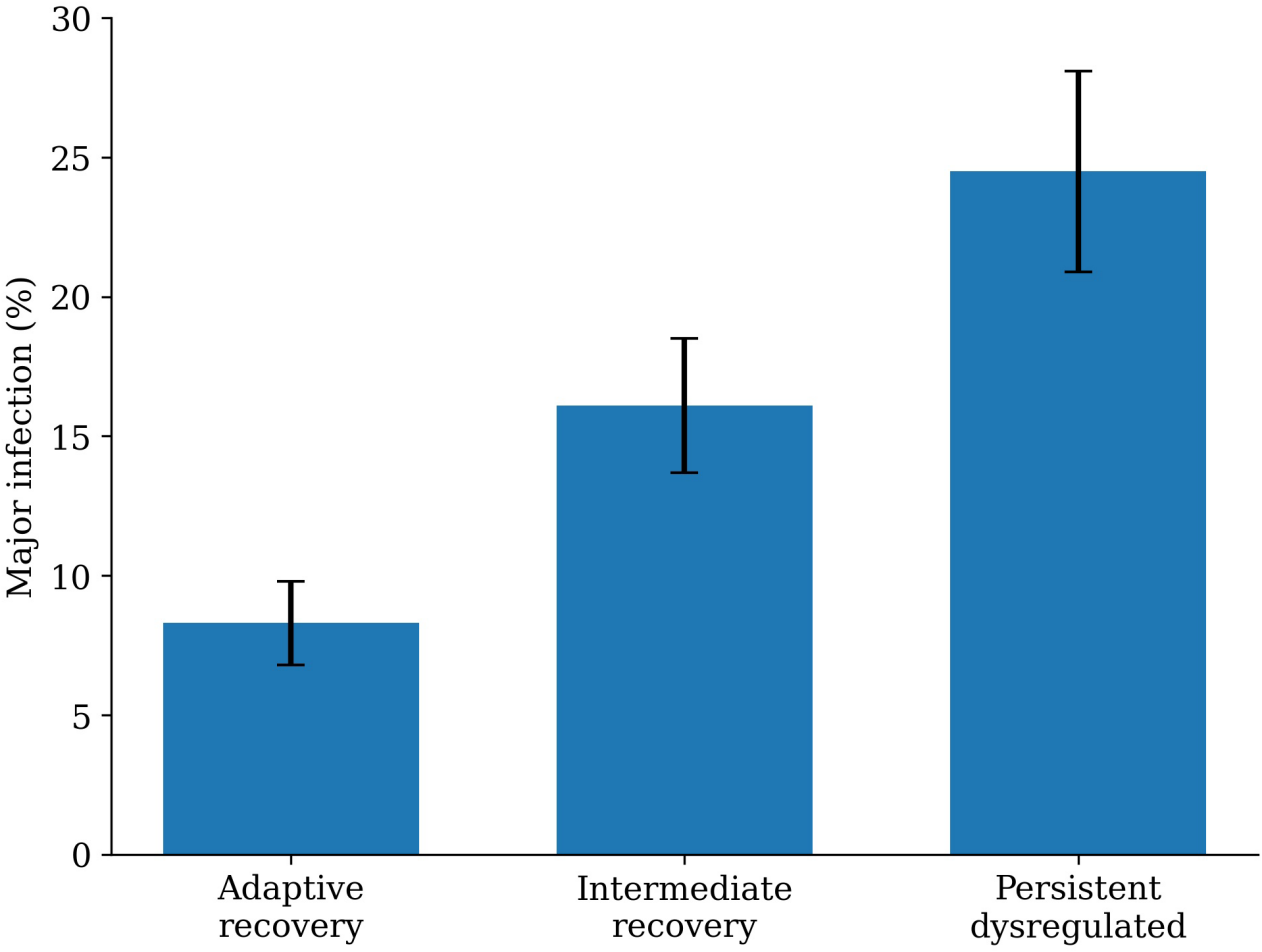
Major postoperative infection according to immune trajectory class. Proportion of patients with major postoperative infection within 30 days according to perioperative immune trajectory class. Error bars represent 95% confidence intervals for the proportion in each class. Major infection occurred in 107 of 1,290 patients (8.3%, 95% CI 6.8%-9.8%) in the adaptive recovery class, 145 of 903 patients (16.1%, 95% CI 13.7%-18.5%) in the intermediate recovery class, and 133 of 543 patients (24.5%, 95% CI 20.9%-28.1%) in the persistent dysregulated class. The between-group difference was significant on chi-square testing (*P <* 0.001).

Major postoperative infection occurred in 385 of 2,736 patients (14.1%) overall. The risk increased progressively across trajectory classes, affecting 107 of 1,290 patients (8.3%) in the adaptive recovery class, 145 of 903 patients (16.1%) in the intermediate recovery class, and 133 of 543 patients (24.5%) in the persistent dysregulated class. A similar gradient was observed for pneumonia, 30-day readmission, and prolonged postoperative length of stay, whereas differences in surgical site infection, urinary tract infection, bloodstream infection, and 90-day reoperation were directionally consistent but not statistically significant. Mean postoperative length of stay was 8.7 ± 1.9 days, 10.4 ± 1.9 days, and 12.7 ± 2.3 days across the three classes, respectively. Discharge biomarker sampling occurred at a median of POD8 (interquartile range, POD7-POD10), with modest between-class differences reflecting longer hospitalization in the persistent dysregulated class. Outcome data are presented in **Table 3**, and the graded rise in major infection is visualized in **Figure 3**.

**Table 3:** Postoperative outcomes by immune trajectory class.

Outcome	Adaptive recovery (*n =* 1,290)	Intermediate recovery (*n =* 903)	Persistent dysregulated (*n =* 543)	*P*
Major infection, *n* (%)	107 (8.3)	145 (16.1)	133 (24.5)	<0.001
Surgical site infection, *n* (%)	25 (1.9)	19 (2.1)	19 (3.5)	0.112
Pneumonia, *n* (%)	23 (1.8)	43 (4.8)	31 (5.7)	<0.001
Urinary tract infection, *n* (%)	13 (1.0)	18 (2.0)	8 (1.5)	0.159
Bloodstream infection, *n* (%)	7 (0.5)	9 (1.0)	6 (1.1)	0.343
Sepsis, *n* (%)	1 (0.1)	6 (0.7)	5 (0.9)	0.02
30-day readmission, *n* (%)	53 (4.1)	70 (7.8)	62 (11.4)	<0.001
90-day reoperation, *n* (%)	26 (2.0)	22 (2.4)	18 (3.3)	0.254
Length of stay, days	8.7 ± 1.9	10.4 ± 1.9	12.7 ± 2.3	<0.001
Prolonged length of stay, *n* (%)	91 (7.1)	230 (25.5)	363 (66.9)	<0.001

### 3.5 Multivariable association of immune trajectory class with adverse outcomes

**Figure 4:**
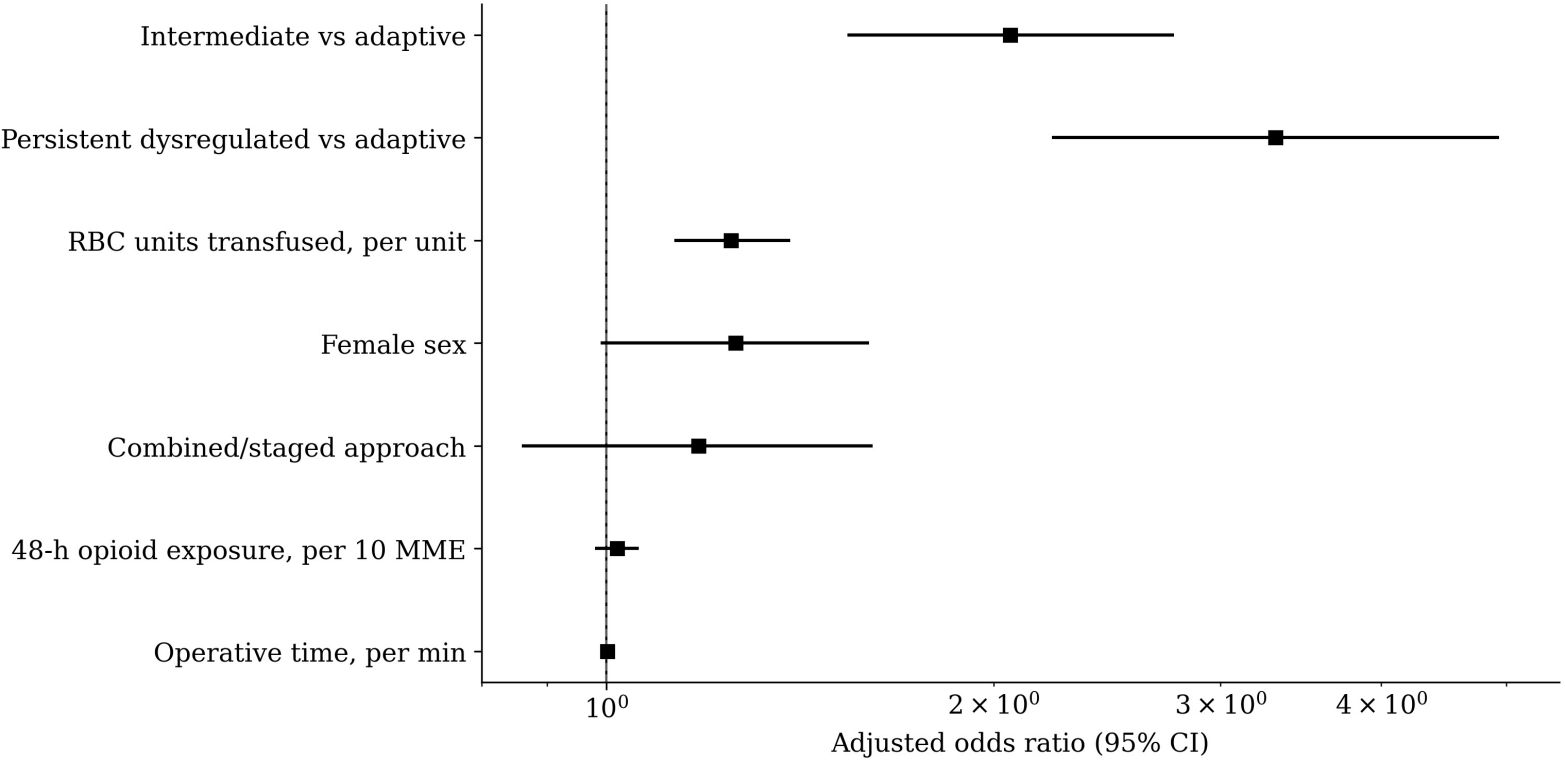
Adjusted predictors of major postoperative infection. Forest plot showing adjusted odds ratios and 95% confidence intervals from the expanded multivariable logistic regression model for major postoperative infection. Relative to adaptive recovery, intermediate recovery was associated with an adjusted odds ratio of 2.06 (95% CI 1.54-2.76), while persistent dysregulated immunity was associated with an adjusted odds ratio of 3.31 (95% CI 2.22-4.94). Each additional unit of red blood cell transfusion was associated with an adjusted odds ratio of 1.25 (95% CI 1.13-1.39). Female sex showed a modest association with major infection (aOR 1.26, 95% CI 0.99-1.60), whereas combined or staged surgical approach and 48-hour opioid requirement were not independently associated with infection. The discrimination of the full model, assessed by the area under the receiver operating characteristic curve, was 0.668, indicating moderate predictive ability.

After adjustment for demographic, deformity-related, operative, and perioperative-management covariates, immune trajectory class remained independently associated with major postoperative infection and prolonged length of stay. Compared with adaptive recovery, intermediate recovery was associated with more than twofold higher odds of major infection, whereas persistent dysregulated immunity was associated with a 3.31-fold increase in odds after adding surgical approach and opioid exposure to the model. For prolonged length of stay, the magnitude of association was stronger, with adjusted odds ratios of 3.71 for intermediate recovery and 17.84 for persistent dysregulated immunity. Red blood cell transfusion was independently associated with both major infection and prolonged hospitalization (35, 36). Female sex showed a modest positive association with major infection in the fully adjusted model, but the trajectory-by-sex interaction was not significant (P for interaction = 0.42), and class-specific associations were directionally similar in females and males. Combined or staged surgical approach and 48-hour opioid requirement did not materially change the trajectory estimates. In the early-window sensitivity model excluding discharge biomarkers, the association with major infection remained directionally consistent for intermediate recovery (aOR 1.88, 95% CI 1.39-2.55) and persistent dysregulated immunity (aOR 2.79, 95% CI 1.86-4.19). The area under the receiver operating characteristic curve was 0.668 for the infection model and 0.824 for the prolonged length-of-stay model, indicating moderate discrimination for infection and stronger discrimination for prolonged hospitalization. Full multivariable results are shown in **Table 4**, and the main adjusted effects for major infection are displayed in **Figure 4**.

**Table 4:** Multivariable logistic regression models for major postoperative infection and prolonged length of stay.

Variable	Major infection, aOR (95% CI)	P	Prolonged length of stay, aOR (95% CI)	*P*
Intermediate recovery vs adaptive recovery	2.13 (1.60-2.84)	<0.001	3.92 (2.97-5.17)	<0.001
Persistent dysregulated vs adaptive recovery	3.54 (2.40-5.21)	<0.001	19.19 (13.40-27.48)	<0.001
Neuromuscular vs idiopathic etiology	1.01 (0.74-1.36)	0.967	1.07 (0.81-1.40)	0.644
Syndromic/other vs idiopathic etiology	1.00 (0.68-1.46)	0.99	1.20 (0.85-1.69)	0.292
Age, per year	0.99 (0.97-1.01)	0.311	1.00 (0.98-1.02)	0.79
Female sex	1.29 (1.02-1.64)	0.035	1.05 (0.85-1.30)	0.674
BMI, per kg/m²	1.04 (1.00-1.08)	0.079	1.01 (0.98-1.05)	0.472
Cobb angle, per degree	1.00 (0.99-1.01)	0.9	1.00 (0.99-1.01)	0.806
Revision surgery	0.97 (0.69-1.35)	0.844	1.64 (1.22-2.19)	0.001
Osteotomy	0.98 (0.77-1.25)	0.896	1.55 (1.25-1.93)	<0.001
Combined or staged reconstruction	1.18 (0.86-1.61)	0.349	1.31 (0.99-1.72)	0.057
Operative time, per min	1.00 (1.00-1.00)	0.024	1.00 (1.00-1.00)	0.154
RBC units transfused, per unit	1.27 (1.15-1.41)	<0.001	1.21 (1.10-1.33)	<0.001
Perioperative steroid exposure	0.94 (0.75-1.17)	0.569	0.83 (0.67-1.02)	0.07
48-hour opioid exposure, per 10 MME	1.02 (0.98-1.06)	0.276	1.05 (1.01-1.09)	0.012
Preoperative hypoalbuminemia	1.00 (0.76-1.31)	0.983	0.98 (0.76-1.26)	0.85

When ICU admission was added as an additional postoperative-severity covariate, the association between trajectory class and infection was attenuated but remained present for intermediate recovery (aOR 1.93, 95% CI 1.43-2.61) and persistent dysregulated immunity (aOR 2.94, 95% CI 1.95-4.43). The findings therefore appear partly explained by surgical and physiological severity but not fully accounted for by the measured perioperative variables.

## 4 Discussion

This large, longitudinal cohort study of 2,736 patients undergoing major spinal deformity correction provides a framework for describing perioperative immune heterogeneity. We identified three distinct immune trajectory patterns using latent class mixed modeling: an adaptive recovery class, an intermediate recovery class, and a persistent dysregulated immunity class. The dysregulated pattern, present in nearly one-fifth of patients, was characterized by sustained lymphopenia, a heightened and prolonged neutrophil-to-lymphocyte ratio (NLR) and interleukin-6 (IL-6) response, lower monocyte HLA-DR expression, lower CD4/CD8 ratio, a stronger C-reactive protein (CRP) response, and delayed albumin recovery. These patterns were associated with clinically meaningful endpoints, including major postoperative infection and prolonged hospitalization, after adjustment for measured surgical burden and perioperative-management variables. However, the trajectories should not be interpreted as purely independent biological endotypes. They likely integrate baseline host vulnerability, scoliosis etiology, operative stress, transfusion exposure, anesthetic and analgesic management, and early postoperative physiological severity.

The lymphopenic nadir and its recovery pattern emerged as a central differentiator among phenotypes. Postoperative lymphopenia is a well-documented consequence of surgical stress (16), but its depth and duration appear to carry prognostic weight. Our dysregulated class exhibited lower absolute lymphocyte counts persistently, aligning with studies linking postoperative lymphopenia to increased infection risk in cancer surgery (37) and poorer survival in gastric cancer (38). The failure of lymphocyte counts to recover promptly may reflect a stronger or more prolonged surgical stress response, unmeasured baseline vulnerability, early infectious complications, or a combination of these factors. In contrast, the adaptive recovery class demonstrated a more transient dip and swift rebound, a pattern associated with better outcomes in other contexts, such as recovery predicting graft function after renal transplantation (39). The neutrophil-to-lymphocyte ratio, a readily accessible composite marker, provided further discrimination. While an elevated NLR is a recognized marker of systemic inflammation and poor prognosis in various surgical and oncologic settings (40–42), our data extend this by demonstrating that the trajectory of NLR is phenotypically distinct. The dysregulated class sustained a high NLR, reflecting both neutrophilia and lymphopenia, a combination linked to adverse outcomes in cardiac (43) and cytoreductive surgery (44). An NLR exceeding 8.5 on postoperative day 3 has been proposed as a cutoff for excluding complications like pancreatic fistula (45); our phenotype-based approach contextualizes such static thresholds within a dynamic patient journey.

The inflammatory cascade, measured through IL-6 and CRP, showed different activation and resolution profiles. IL-6, a key early mediator of the surgical stress response (16, 46), peaked higher and remained elevated in the dysregulated class. This aligns with evidence that higher IL-6 levels correlate with greater surgical stress (46), postoperative complications (47), and unresolved cognitive dysfunction (48). In this retrospective cohort, persistent IL-6 elevation cannot be assigned to a single mechanism; it may reflect greater tissue injury, transfusion exposure, early occult infection, delayed recovery, or expected physiological response after extensive reconstruction. Similarly, CRP kinetics differed substantially. While a postoperative CRP rise is expected, its magnitude and decline are informative (49, 50). A secondary rise or failure to decline after day 3–5 is a recognized red flag for infectious complications like anastomotic leak (51, 52) or surgical site infection (49). Our dysregulated phenotype exhibited this pattern, a robust CRP response with delayed resolution, which correlated with their higher infection rate. This supports the clinical utility of serial CRP monitoring, though its standalone value for early infection detection after major spinal surgery may be limited (53). Monocyte HLA-DR expression, a marker of innate immune competence, was lower in the dysregulated class (54). We therefore use the term immune suppression cautiously: lower mHLA-DR is compatible with impaired antigen-presentation capacity, but the present data cannot establish clinical immunoparalysis without functional immune testing.

Shifts in adaptive immunity, indicated by a lower CD4/CD8 ratio, and metabolic-integrative markers like albumin, completed the phenotypic picture. A decreased CD4/CD8 ratio suggests a relative increase in cytotoxic or suppressor T-cell populations, potentially indicating an immunosuppressive state (55). Delayed albumin recovery is particularly noteworthy. While preoperative hypoalbuminemia is a known risk factor, postoperative albumin dynamics (ΔAlb) are gaining recognition as a powerful prognostic indicator (56–58). A significant postoperative drop in albumin reflects not only nutritional stress but also capillary leak and sustained inflammation, acting as an integrative marker of physiological reserve and recovery capacity. The strong association between the dysregulated phenotype, with its delayed albumin recovery, and prolonged hospitalization underscores the clinical relevance of this simple measure. It moves beyond a static preoperative snapshot to capture the dynamic metabolic cost of surgery and the body’s ability to restore homeostasis.

The mechanisms linking the dysregulated immune trajectory to adverse outcomes are likely multifactorial (59). The observed biomarker pattern is compatible with an amplified and prolonged surgical stress response (16, 60), but it may also partly reflect differences in deformity severity, operative technique, transfusion exposure, ICU care, and early postoperative complications. The initial pro-inflammatory surge (high IL-6, NLR) may be followed by compensatory anti-inflammatory features, including lymphopenia and lower mHLA-DR, creating a possible window of vulnerability for pathogens (61). At the same time, these changes may represent expected physiological responses after extensive spinal reconstruction rather than pathological immune failure in every patient. The persistent inflammatory milieu, evidenced by high CRP and IL-6, can impair wound healing, promote insulin resistance, and exacerbate catabolism, contributing to fatigue and functional decline (16). This study did not examine long-term outcomes, and causal inference should therefore remain cautious.

### 4.1 Clinical implications

From a clinical standpoint, the findings support dynamic immune monitoring as an adjunct to, rather than a replacement for, conventional postoperative assessment. Because the full six-time-point trajectory uses discharge data, it is best viewed as a descriptive and risk-stratification framework. The early-window analysis suggests that preoperative through POD3 data may already identify patients trending toward a higher-risk profile, but this requires prospective validation before being used for decision-making. In practice, patients with persistent lymphopenia, rising NLR or IL-6, delayed CRP resolution, and low albumin by POD3 could reasonably trigger closer pulmonary and wound surveillance, review of antimicrobial indications, nutritional optimization, and more cautious discharge planning (62). The independent association of red blood cell transfusion with both infection and prolonged stay reinforces the importance of rigorous blood conservation strategies (63–65). Integrating immune phenotyping into Enhanced Recovery After Surgery (ERAS) pathways (66, 67) may ultimately personalize care, but intervention thresholds and clinical utility remain to be defined.

### 4.2 Strengths and limitations

The primary strengths of this study include its large sample size in a homogeneous surgical population, the dense longitudinal sampling of multiple immune parameters, and the application of advanced statistical modeling (LCMM) to uncover latent phenotypes rather than imposing arbitrary cut-offs. This data-driven approach captures the complexity and interplay of the immune response more holistically than analyzing biomarkers in isolation (42, 68). By linking the phenotypes to hard clinical endpoints with strong adjusted effect sizes, we demonstrate their tangible relevance for postoperative management and resource utilization.

Several limitations must be acknowledged when interpreting the results. As a single-center retrospective study, the findings are susceptible to selection bias and may not be fully generalizable to other institutions, surgical teams, anesthesia protocols, or different types of major surgery (69, 70). Despite multivariable adjustment, residual confounding remains likely (71). In particular, immune trajectories differed according to operative burden, transfusion requirement, ICU admission, and scoliosis etiology; therefore, they may partly represent the physiological footprint of more complex surgery rather than distinct pre-existing immune biology. We added surgical approach, steroid exposure, and opioid requirement to the adjustment strategy, but detailed medication timing, intraoperative hemodynamics, antibiotic changes, and functional immune assays were not uniformly available (72–74). Discharge biomarker timing was not identical for all patients, and longer hospitalization in higher-risk patients may have influenced the discharge-time trajectory. The early-window sensitivity analysis mitigates but does not eliminate this concern. Procalcitonin was not incorporated because serial measurements were incomplete and clinically indicated rather than protocolized. The lack of external validation is a significant constraint; the identified phenotypes and their associated risk coefficients require confirmation in independent, prospective cohorts. Finally, the immune measurements, while comprehensive, represent a systemic snapshot and may not fully reflect local tissue-level immune processes at the surgical site.

### 4.3 Future directions

Future research should directly address the limitations. A critical next step is the external validation of the immune phenotypes in multi-center prospective studies of spinal deformity and other major surgical cohorts. Prospective validation would also allow for the development and testing of real-time clinical decision algorithms based on early postoperative biomarker trends. Mechanistically, deeper profiling is needed to understand the drivers of the dysregulated phenotype (75). This could involve multi-omics approaches, transcriptomic, proteomic, and metabolomic analyses, on serial samples to delineate the upstream signaling pathways and downstream effector functions (47, 60, 76). Exploring the role of the gut microbiome and endothelial function in shaping the immune trajectories presents another fertile avenue. From a translational perspective, interventional trials are warranted. Can early immunomodulatory strategies, such as targeted anti-cytokine therapies (e.g., IL-6 blockade) in select high-risk patients (77, 78), immunonutrition, or personalized glycemic and hemodynamic management, attenuate the dysregulated phenotype and improve outcomes? Investigating the long-term consequences of the perioperative immune trajectories on functional recovery, quality of life, and in oncologic cases, cancer recurrence, would be of great value (63, 79). Ultimately, the goal is to evolve perioperative care from reactive complication management to proactive immune system stewardship, leveraging dynamic phenotyping to deliver truly personalized and precision medicine.

## 5 Conclusions

In patients undergoing surgery for severe scoliosis, perioperative immune recovery is not uniform but segregates into distinct longitudinal patterns with clinical relevance. Patients with persistent dysregulated immunity show deeper and more sustained lymphopenia, greater postoperative inflammatory activation, higher IL-6 and neutrophil-to-lymphocyte ratio levels, lower monocyte HLA-DR expression, and delayed recovery of albumin and cellular immune balance, whereas patients with adaptive recovery demonstrate a more favorable return toward immunologic homeostasis. This immune trajectory classification is associated with adverse short-term outcomes, particularly major postoperative infection and prolonged hospitalization, even after adjustment for measured operative complexity, surgical approach, transfusion burden, steroid exposure, and opioid requirement. The findings suggest that postoperative risk after severe scoliosis correction reflects both the magnitude of surgical stress and the pattern and persistence of host immune response across the perioperative course. Longitudinal immune-trajectory profiling may provide a useful framework for identifying vulnerable patients and refining postoperative monitoring, but prospective multicenter validation and assessment of modifiable interventions are needed before routine clinical implementation.

## Data Availability

The raw data supporting the conclusions of this article will be made available by the authors, without undue reservation.
